# Facts and Philosophy of Dental Progress. No. 3

**Published:** 1867-11

**Authors:** 


### ARTICLE III.

### *Facts and Philosophy of Dental Progress. No.* 3.

By Professor Austen.

In our last paper it was stated that mechanism and manual dexterity form the distinctive elements of dentistry, as a branch of the Art of Healing—that the Dentist is, in nearly every department of his art, necessarily a mechanic. It is a grave error therefore to undervalue this essential element, and suppose that any one may be trained for dentistry as readily as for medicine or surgery: but perhaps a greater error is the very prevalent idea, that every ingenious youth is a distinguished dentist in embryo.

Protest against this mistake is the more necessary here, as all our illustrations of dental progress will be drawn from its mechanical side; since it is because of these that its progress has been so rapid—superadding the benefits of mechanical invention to the aid derived from physiological discovery.

From the moment of the patient's entrance into the office, you begin to appreciate the assistance rendered by improvements in dental mechanism. The chair may be like any other, with the simplest form of *rest* for tedious operations. And it will not be denied that, in such chairs, much work has been done of unsurpassed excellence. But in the modern dental chair, whilst there is frequently unnecessary and complicated contrivance, there is undeniable advantage in the facility of placing the patient in various positions with ease and comfort. Restlessness on the part of patient, and weariness on the part of operator, are serious obstacles, and whatever lessens them is not to be despised, because the aid comes from the chair maker.

The old practitioner, who began with a simple arm chair and knows that he has learned to do good work in it, is prouder of his work perhaps, than if done in a chair, which might have spared him some dorsal twinges of his own and some restless turnings of his patient. Attachment to the "old arm chair" is very excusable: but becomes weakness, where it refuses to spare others the experience necessary to make it a "dental chair."

Complicated chairs, jeweled instruments, beautiful rooms and light artistically arranged, do not guarantee good dentistry: but they certainly (the jewels excepted) are not hindrances to the exercise of skill. They are often the pharaphernalia of incompetent quackery: but quite as much bad work has been done in dingy rooms, with dirty surroundings and few and imperfect appliances. It is painful to enter an office, where the only deficiency is in the operator; but scarcely less painful to see one, whose skill has overcome difficulties, persisting in the contest.

These remarks apply not to chairs alone, which rank among less important improvements, but to all the means offered by inventive genius, for the more perfect developement of manual skill. Long practice makes a defective instrument useful and possibly indispensible to one; but another, commencing with the improved implement, is spared much waste of effort, and can, perhaps with less skill, produce the same results.

How far one is called upon to give up accustomed tools, methods, or materials, is not easily decided. Certainly there is no practice more fatal to success than the too-frequent one of perpetual change. No mechanism insures good results, apart from experienced manipulation. The operator, who is always praising the latest invention, may safely be set down, as one who has an imperfect knowledge of any. It would be amusing were it not so painful, to hear one of these experimental dentists asserting five times, within twice as many years, that "now since adopting this plan, my operations never fail." Constant to no one method, he is successful in none and his skill is far more wasted than is that of the old fogey, who will accept no "new fangled" help.

Dwinnelle's success with chrystal gold cannot tempt Maynard to give up non-adhesive foil; because such foil, under his hand, gives a *perfect* result, and more cannot be required: because also, he can do with foil what, without long experience, he could not with the sponge; and because the older material has stood the test of time. But all are not Maynards in the use of an old, or Dwinnelles in the use of a new material.

Long and *exclusive* use of a method or instrument is essential to the complete developement if its merits and dental progress is equally served by him who persists in the old, as by him who identifies himself with the new. Atkinson's little mallets will probably hammer out startling facts and possibly upset some old ideas in physiology. We may even anticipate the time when miniature "Nasmyth Steam Hammers" will do all the work of filling cavities, prepared by diminutive "Steam Excavators." Yet I think manufacturers of the old-timed foil will continue to have customers and am not sure that they will not be called upon occasionally to open again some old account. When you wish, gentlemen to try a new string to your dental bow, never throw away the old one. Possibly the new one may prove stronger; but the old strings have been tried and have this merit, that they improve with use. Above all, take care that, in the recital of your experiences, you do not shoot with the "longbow." It is a weapon which has done vast mischief to Dental Science.

An important question arises here, as to the fitness of a specialist to teach. It would be irrelevent to the subject in hand to enter fully into it: but it may be stated briefly —the specialist is the best possible *teacher* of his speciality; the worst possible *adviser* as to its relative value. In my papers on Dental Education, this point will be fully considered. As regards Dental Progress, the specialist is a necessity. He developes truths which a judicious eclecticism disseminates and makes generally useful.

Returning once more to the chair, in which I place myself as patient—I may be a little indignant with the operator, who is regardless of my bodily comfort during a tedious operation : but if I find him adjusting too many screws and cranks and slides, I begin to have an uncomfortable suspicion that he is more "fussy" than skilful. If I see him take from a dirty drawer of files, teeth and other miscellanies an old rusty key, I tremble with fear and disgust. But if I see him hesitatingly turning over his polished collection of fifty or more "alveolas,"* I think I would rather try the blood-stained "key," or else subside into the unconsciousness of nitrous oxide.

Too much apparatus excites distrust ; too little impedes skill. It is a rule in Art to use the fewest tools ; but these should be the best. It is another rule to have them of simplest form. The more complicated an instrument, the more limited is its usefulness. The operator's cabinet should contain a few simple tools for daily use ; others again, including the more complicated, for exceptional cases and modes of practice. He should avail himself of all appliances which give convenience and facility of operating and may very properly consult the comfort of his patient. But he must not become the slave to any of these appliances, or insult his power of resource, by having separate instruments for every emergency. One will have six tools with the same point, but differently curved ; while Maynard will use but one, curving it at the moment as the occasion may require. This one instru-

*Will the Commmittee on Instrumental nomenclature, inform the writer why the final r is so unnecessarily retained in the molar forceps? Why not call them "Molas?" There is no wish to make a mountain out of a mole hill; but why, in search of a trade mark, contort alveolus or alveoli into an impossible alveola, when the word "molar" so naturally suggests the analogous adjective "alveolar."

ment, with the ability to adapt it, is worth six times the other six. Again, he will accomplish with the napkin all that the best saliva pump can do ; and yet this is an excellent instrument, for comparatively few can manage the napkin.

These simple illustrations, borrowed from an eminent practitioner, are given to show the necessity for varieties of apparatus, adapted to individual peculiarities, or deficiencies. It is scarcely fair to measure the wants of the average, by the requirements of the most skilful. Hence every operating case contains more than any operator can use with advantage ; but not more, if judiciously chosen, than is necessary to enable him to select the daily tools which best suit his hand. In astronomical observations there is a correction for "personal error." Now, as in the observatory, a given observer will take the moment of transit invariably a little too soon or too late ; so every artist or mechanic will have some peculiarities in the handling of tools, calling for differences in their number and shape.

That the best possible fillings have been made with old-fashioned tools and materials and by time-honored methods, is undeniable, and continuance in their use is imperative, until their substitutes have stood the test of experience. It is to the honor of Dentistry that so many are conscientiously searching for better, and the profession will be greatly the gainer, whatever the success of any single experiment.

In this search for the best, the older practitioner should be careful, how he abandons the *proven* good, for the *untried* better : and the younger practitioner has need to be careful in the choice of advisers and teachers, careful how he adopts too many methods, and careful how he cramps his inventive resource by an accumulation of tempting machinery.

